# Collectanea Medica: Consisting of Anecdotes, Facts, Extracts, Illustrations, &c.

**Published:** 1822-03

**Authors:** 


### COLLECTANEA MEDICA:

CONSISTING OF

### ANECDOTES, FACTS, EXTRACTS, ILLUSTRATIONS, &c.

*Relating to the History or the Art of Medicine, and the Collateral Sciences.*

Floriferis, ut apes, in saltibus omnia libant,

Omnia nos, itidem, depascimur aurea dicta.

---

Art. I.—*On Artificial Mineral Waters; with some Remarks on Artificial Light.* By Samuel Morey, of Oxford, New Hampshire, U. S. (From Silliman's American Journal of Science and Arts, vol. iii.)

THE object of the following thoughts and experiments is to furnish, at a cheap rate, an abundant supply of light for many purposes, and also to multiply fountains, such as those of Seltzer, Saratoga, &c. at such an expence, and in such quantities and situations, that the calls and necessities of every one may be conveniently satisfied.

It is well known that the vapour of water, in passing through ignited charcoal, is decomposed. If the product is made to pass through water, the carbonic acid gas is absorbed, and the hydrogen is so far separated as to be at command for furnishing light, filling balloons, or other purposes. If water be composed of 85 parts of oxygen and 15 of hydrogen; and carbonic acid gas, of about 28 of carbon in the 100; one pound of charcoal, and not far from four of water, ought to give something like 250 gallons of carbonic acid gas, or enough to supply that quantity of as nearly strongly impregnated mineral water as is to be found in nature; and near 500 gallons, or about 75 cubic feet of hydrogen gas, something like equal to 25 pounds, or six wax candles burning twenty-five hours, for affording light. Some very trifling addition of spirit of turpentine, or other substance, will be required to make the flame white. These gases, as formed, may be forced into the aqueduct of limited quantity of water, to be conveyed along with it to some desirable situation, to be let out for use. The quantity of water let out will depend on the quantity of mineral water wanted. If a flame be applied to the surface of these fountains, the hydrogen gas takes fire, and burns on the surface of the water; thereby, perhaps, exposing some of nature's hidden operations.

In this way, towns, cities, and manufactories, may be, for ought that yet appears, supplied with a pleasant healthy drink, and at the same time, and from the same materials, abundantly lighted. There must be an addition of some kinds of fuel, to preserve the red heat of the charcoal, and to evaporate the water. But that will be trifling; especially if it be a fact, as I fully believe, that carbonic acid gas, in forming, always gives out, instead of absorbing, heat. If so, it is, at least, one source of animal heat. As yet, my attention, so far as my health would permit, has been directed chiefly with a view to come at an easy mode of furnishing mineral springs, by the disengagement of the carbonic acid from marble by the stronger acids. For that purpose, and others, I had a small cool spring of water brought in a wooden aqueduct about 150 yards, having a descent of fifty feet, all the way on an inclined plane; so that the water should, in no instance, fill the bore of the logs, and obstruct the passage of the gas or gases, if turned into the aqueduct with the water, or forced in at the bottom.

I found no difficulty in filling the aqueduct with water, so as to give a pressure or head equal to thirty or forty feet perpendicular. Very fine streams of water, issuing under any thing like this head, and coming in contact, at nearly a right angle, with a plane surface, were converted into a spray, as fine, much of it, as mist. When this mist or spray was forced into an atmosphere of pretty-strongly compressed carbonic acid gas, it became suitably impregnated with the gas, so as to form a very pleasant drink. To facilitate the impregnation, and to prevent a possibility of any of the sulphuric acid being retained by the water, and for other purposes, I filled the vessel (a common three-gallon stone jug answers well,) about two-thirds or three-fourths, with grains of the marble, about the size of large peas; the spray falling on these, passing slowly from one piece to another, gives more time, and presents an immense surface to the gas. In this way, water, under a pressure of twenty or thirty feet head, or much less, issuing at the rate of about two half-pint tumblers per minute, flows in pure spring water at the top, and flows out nearly, or perfectly, saturated at the bottom; as much so, nearly, as any known natural spring. By adding one vessel to the top of another, or increasing the length and increasing the pressure, any desired quantity of the gas may be added to the water. Indeed, any part, or the whole length, of the aqueduct, may be filled with these fragments, and with those of iron. The gas and water evidently dissolve more or less of the lime and iron. Carbonate of soda, or any other substance which renders the water more healthy or useful, may be added in any desired quantity. The quantity of water that flows in may be regulated or stopped, so as to meet, very exactly, the call or demand there shall be for the mineral water. The overplus of the spring water is applied to keep the vessels and mineral water cool. This mineral water, with a portion of the gas, may be continued into an aqueduct, and discharged into the bottom of a box, in which they will rise up for use, with the same bubbling appearance as those of the natural mineral waters. Some of the marble, which I use, appears to contain much sulphuretted hydrogen, and some iron pyrites. If the mineralized water stands a day or two on these fragments of lime, it gives the water as strong a smell and taste of this gas, probably, as any natural spring in the world; and there is an appearance of iron being deposited from this water, by standing in a glass vessel. How easy then to produce, at one establishment, from a single spring of pure water, our choice of any number of mineral waters; and those as good as any to be found in nature, or possibly better.

If the vinous fermentation should be adopted for procuring the carbonic gas, a flood of it may be procured from many sources, not only without expence, but even at a profit.

But, in those situations where these waters will be most wanted, and where they are much the most useful, I know of no reason why an abundant supply of the gas, ready formed, may not always be had, and that without expence: I allude to breweries. At as many of these as is necessary, (where there is steam or other power,) it will be best to avail ourselves of a forcing pump, to drive the gas, as formed and purified, or from reservoirs, into suitable pipes, with a sufficient quantity of water, to be conducted to some pleasant, suitable situation, far enough to have the water properly impregnated, and then to be let out for use. If a greater pressure than what the ground naturally affords be wanted, it is only to add, at the outlet, a valve sufficiently loaded. A small fountain may receive the water and overplus gas, above the valve, or they may be drawn below.

It appears necessary that, where the gas is disengaged from limestone, the vessel containing that and the diluted acid should in some way be agitated, so as to shift the position of the pulverized marble. The best mode I have tried, as yet, is to cause the vessel to revolve (say a stone jug,) on an axis, and end over end; and to have a portion of the marble coarse. As the sulphate of lime forms on the surface of these lumps, by their friction in rolling over each other, it appears to be worn off, presenting continually fresh surface for the acid to act upon, and thereby continuing very regularly its operation, ever so long. These vessels may be made to revolve (very slowly, if necessary,) by weights, springs, or a parapet of the water, or may occasionally be moved by hand. The gas presses out at one end of this axis. A very cheap, quick, and agreeable mode of preparing the water, where we have, or have not, an aqueduct or head to resort to, is to take four or five, or more, decanters, (say of the capacity of a quart;) fill them with fragments of marble; set them in a row, or other form, each with a good cork; let a tin tube screw upon the axis to receive the gas, and press pieces of cork, or other stuffing, to the vessel, to make it tight round the axis. This tube, near the other end, is of a conical form, and turned down at a right angle, far enough to be inserted through the cork of the first bottle: another tube, turned down at each end, so that one leg shall pass through the cork also to the bottom of the first decanter, and the other through the cork of the second decanter; and so on with as many as are to be used. A small reservoir is to be placed as many feet above as convenient, and the first vessel, with a small pipe leading down and through the first cork, with an opening at the lower end, about one-thirtieth of an inch in diameter. A quantity of pulverized lime and water may be put into the revolving vessel; then more water, containing a small quantity of clay or sulphate of lime. This will be deposited on the lime, so as to prevent the action of the acid when poured in, which may now be added, and the vessel corked. It is now ready for use.

Put cool water into the reservoir; turn the vessel, moderately, a very little, if the gas is not already forming fast enough. The water strikes on the first fragments of stone, is thrown over them, and passes over the surface, from one to another, to the bottom, where it is continually taken up by the tube, with a portion of the gas, and discharged just below the cork of the second vessel; or thrown out, in a spray, by and with the gas; or else discharged at the lower end of the tube in bubbles; thereby furnishing a carbonic gas within and without, with an almost infinitely thin film of water between, to absorb it on each side. By continuing the same process, it passes the whole, let the number be ever so many. At the last one it is thrown out through a stop-cock, as at common soda-water establishments, or flows out in a continued stream with the gas. It may be as well to fill the first decanter, or even all of them, with water, and discharge them into the last one, by the gas, before the water is let in from the reservoir above. These glass vessels may be placed in a trough to keep them cold: this can stand on a table or side-board, where the domestics, or other members of the family, may, at any time, furnish a bottle or bottles of the mineral water when wanted.

If tin-plate tubes will answer, it is but a short day's work to prepare this apparatus; which may afford sixty or 120 tumblers per hour, with half-a-dozen decanters. By increasing their number or size, any given quantity, in this way, can be constantly or occasionally supplied. One pound of sulphuric acid, if I mistake not, ought to furnish gas enough to saturate sixty or seventy gallons, or not far from 1000 tumblers of water. Experiments, I think, justify me in saying, we may expect not far from half this quantity, practically. I can but hope, and do believe, if owned and properly managed by corporations and companies, it may and will do much, very much, towards annihilating the use of ardent spirits, as well as be the means of saving many, annually, from an untimely death by drinking cold water; to say nothing of its usefulness in other respects.

When currents of this gas, conveniently situated, are found issuing naturally from the earth, they may be turned to account; as, for instance, at the Grotto del Cane, two miles from Naples. If the gas from this grotto were received into an aqueduct, and carried in the direction of Naples, or in any other, until a supply of water could be added, the current might be continued until the gas and water were perfectly united; and then they might be let out for use in any situation most agreeable, or most conducive to the comfort and convenience of that city or country.

P.S.—From an ounce of sulphuric acid, I practically obtain (I may say, without trouble,) about twenty tumblers of as pleasant drink as I could wish for.

It is a great luxury; and I have no doubt that it has been of much service to my health. The sulphate of lime, here, is worth six or eight times what the ground marble costs.

**Art. II.—*Unparalleled Instance of Suicide, by voluntary Starvation.***

Luc Antoine Viterbi was condemned to death as an accomplice in the assassination of Frediani, a crime which he denied to the last moment, and appealed against a sentence passed upon him by a court composed of his personal enemies.

Towards the end of November, Viterbi, (knowing his condemnation, and being confined in the prison of Bastia,) resolved to die. To effect his purpose, he abstained from food for three days, and then ate voraciously, and to a forced excess, in the hope that, after fasting so long, he should thereby put an end to his existence. Nature deceived him, and, on the 2d of December, he determined to starve himself to death. From that day nothing could subdue this terrible resolve; although Viterbi, who had already sustained two dangerous attacks of illness, did not expire until the night of the 21st of that month.

During the three first days, Viterbi, as was the case when he made the first attempt, felt himself progressively tormented by hunger, and did not endure these early sufferings with less courage than he had shown on the former occasion. Under these circumstances, a report was made to the public Minister, who ordered bread, water, wine, and soup, to be taken daily to his cell, and placed conspicuously in view. No debility was manifest during these three days, no irregular muscular movement was remarked, his ideas continued sound, and he wrote with his usual facility, but took no nourishment.

From the 5th to the 6th, to hunger insensibly succeeded the much more grievous suffering of thirst, which became so acute, that on the 6th, without ever deviating from his resolution, he began to moisten his lips and mouth occasionally, and to gargle with a few drops of water, to relieve the burning pain in his throat; but he let nothing pass the organs of deglutition, being desirous not to assuage the most insupportable cravings, but to mitigate a pain which might have shaken his resolution. On the 6th, his physical powers were a little weakened; his voice was, nevertheless, still sonorous, pulsation regular, and a natural heat equally extended over his whole frame. From the 3d to the 6th, he had continued to write, at night several hours of tranquil sleep seemed to suspend the progress of his sufferings, no change was observable in his mental faculties, and he complained of no local pain.

Until the 10th the thirst had become more and more insupportable: Viterbi merely continued to gargle, without once swallowing a single drop of water; but in the course of the 10th, overcome by excess of pain, he seized the jug of water, which was near him, and drank immoderately. During the last three days, debility had made sensible progress, his voice became feeble, pulsation had declined, and the extremities were cold. Viterbi, however, continued to write; and sleep, each night, still afforded him several hours' ease.

From the 10th to the 12th the symptoms made a slight progress. The constancy of Viterbi never yielded an instant; he dictated his journal, and afterwards approved and signed what had thus been written agreeably to his dictation. During the night of the 12th, the symptoms assumed a more decided character, debility was extreme, pulsation scarcely sensible, his voice extraordinarily feeble, the cold had extended itself all over his body, and the pangs of thirst were more acute than ever. On the 13th the unhappy man, thinking himself at the point of death, again seized the jug of water, and drank twice, after which the cold became more severe; and, congratulating himself that death was nigh, Viterbi stretched himself on the bed, and said to the gendarmes who were guarding him, "Look how well I have laid myself out." At the expiration of a quarter of an hour, he asked for some brandy; the keeper not having any, he called for some wine, of which he took four spoonfuls. When he had swallowed these the cold suddenly ceased, heat returned, and Viterbi enjoyed a sleep of four hours.

On awaking (on the morning of the 13th), and finding his powers restored, he fell into a rage with the keeper, protesting that they had deceived him, and then began beating his head violently against the wall of his prison, and would inevitably have killed himself, had he not been prevented by the gendarmes.

During the two following days, he resisted his inclination to drink, but continued to gargle occasionally with water. During the two nights he suffered a little from exhaustion, but in the morning found himself rather relieved. It was then that he penned some stanzas.

On the 10th, at five o'clock in the morning, his powers were almost annihilated, pulsation could hardly be felt, and his voice was almost wholly inaudible; his body was benumbed with cold, and it was thought that he was on the point of expiring.—At ten o'clock he began to feel better, pulsation was more sensible, his voice strengthened, and, finally, heat again extended over his frame, and in this state he continued during the whole of the 17th. From the latter day until the 20th, Viterbi only became more inexorable in his resolution to die.

During the 19th, the pangs of hunger and thirst appeared more grievous than ever; so insufferable, indeed, were they, that, for the first time, Viterbi let a few tears escape him. But his invincible mind instantly spurned this human tribute. For a moment he seemed to have resumed his wonted energy, and said, in the presence of his guards and the gaoler, "I will persist, whatever may be the consequence; my mind shall be stronger than my body; my strength of mind does not vary, that of my body daily becomes weaker."

A little after this energetic expression, which showed the powerful influence of his moral faculties over his physical necessities, an icy coldness again assailed his body, the shiverings were frequent and dreadful, and his loins, in particular, were seized with a stone-like coldness, which extended itself down his thighs.

During the 19th, a slight pain at intervals affected his heart, and, for the first time, he felt a ringing sensation in his ears. At noon, on this day, his head became heavy; his sight, however was perfect, and he conversed almost as usual, making some signs with his hands.

On the 20th, Viterbi declared to the gaoler and physician, that he would not again moisten his mouth; and, feeling the approach of death, he stretched himself on the bed, and asked the gendarmes, as he had on a former day, whether he was well laid out, and added, "I am prepared to leave this world." Death did not this time betray the hopes of a man who, perhaps, since the creation, invoked it with the greatest fervour, and to whom it seemed to deny its cheerless tranquillity.

On the 21st, Viterbi was no more. Until the day of his death this man regularly kept his journal. The delivery of it to his family was refused.—*(From the Corsican Gazette.)*

---

### Art. III.—*Report from the National Vaccine Establishment.*

To the Right Honourable Robert Peel, Principal Secretary of State for the Home Department.

*National Vaccine Establishment, Percy-street, Jan. 31.*

Sir,—Vaccination has now been submitted to the test of another year's experience, and the result is an increase of our confidence in the benefits of it. We are happy to say, that it appears to have been practised more extensively than it was, notwithstanding the influence of exaggerated rumours of the frequent occurrence of small-pox subsequently, on the minds of some persons, and the obstinate prejudices of others, who still continue to adopt inoculation for that disease. The unavoidable consequence of the latter practice is to supply a constant source of infection, and to put the merits of vaccination perpetually to the severest trial.

Of small-pox, in the modified and peculiar form which it assumes when it attacks a patient who has been previously vaccinated, many cases, indeed, have been reported to us in the course of last year, and some have fallen within the sphere of our own observation; but the disorder has always run a safe course, being uniformly exempt from the secondary fever, in which the patient dies most commonly when he dies of small-pox.

For the truth of this assertion, we appeal to the testimony of the whole medical world; and, for a proof that the number of such cases bears no proportion to the thousands who have profitted, to the fullest extent of security, by its protecting influence, we appeal confidently to all who frequent the theatres and crowded assemblies, to admit that they do not discover, in the rising generation, any longer, that disfigurement of the human face which was obvious every where some years since.

To account for occasional failures, of which we readily admit the existence, something is to be attributed to those anomalies which prevail throughout nature, and which the physician observes, not in some peculiar constitutions only, but in the same constitution at different periods of life, rendering the human frame at one time susceptible of disorder from a mere change of the wind, and capable, at another, of resisting the most malignant and subtle contagion. But, amongst the most frequent sources of failure which have occurred, and will for a time continue to occur, is to be numbered that careless facility with which unskilful benevolence undertook to perform vaccination in the early years of the discovery; for experience has taught us, that a strict inquiry into the condition of the patient to be vaccinated,—great attention to the state of the matter to be inserted,—and a vigilant observation of the progress of the vesicles, on the part of the operator,—are all essentially necessary to its complete success.

That less enlightened parents should hesitate to accept a substitute for inoculation, which is not perfect in all its pretensions, and absolutely and altogether effectual to exempt the objects of their solicitude from every future possible inconvenience, does not surprise us; but we cannot forbear to express our unqualified reprobation of the conduct of those medical practitioners, who, knowing well that vaccination scarcely occasions the slightest indisposition,—that it spreads no contagion,—that, in a very large proportion of cases, it affords an entire security against small-pox, and, in almost every instance, is a protection against danger from that disease,—are yet hardy enough to persevere in recommending the insertion of a poison, of which they cannot pretend to anticipate either the measure or the issue, (for no discernment is able to distinguish those constitutions which will admit inoculated small-pox with safety;) and there are some families so dangerously affected by all the eruptive diseases, that they fall into imminent hazard in taking any of them. This remark has a particular application to small-pox,

A family lost its two first-born children of the small-pox, inoculated by two of the most skilful surgeons of the time: nor is it improbable that the parents might have had to lament the loss of more children, under the same formidable disease, if the promulgation of the protecting influence of vaccination had not happily interposed to rescue them from the consequences of a repetition of the fatal experiment. Of their remaining children, one took the small-pox after vaccination, and went through it in that mild and mitigated form which stamps a value upon its resource, as real, in the eye of reason and sound philosophy, as when it prevents the malady altogether.

We have contended, Sir, for this its merits, with all the powers of our understanding, and with all that just and fair pretension to convince others, to which we are entitled by being firmly and sincerely convinced ourselves. Nor shall we relax in our efforts to promote its adoption, but continue to exert the influence which the benevolent designs of Parliament, in establishing this Board, have given us for extending the benefits of this salutary practice.

That the blessing is not yet absolutely perfect, we are ready to admit; but, when we compare it with inoculation for the small-pox, the only alternative, we have no hesitation in stating that the comparison affords an irresistible proof of its superior claims to regard; for we learn, from ample experience, that the number of cases of small-pox, in the safe form which it is found to assume after vaccination, is by no means equal to the number of deaths by inoculation: an evidence quite irrefragable, and, as it appears to us, decisive as to the incalculable advantages of the practice of the first over that of the latter method.

The number of persons who have died of small-pox this year within the bills of mortality, is only 508,—not more than two-thirds of the number who fell a sacrifice to that disease the year before; and as, in our last report, we had the satisfaction of stating that more persons had been vaccinated during the preceding than in any former twelve months, we flatter ourselves that this diminution of the number of deaths from small-pox may fairly be attributed to the wider diffusion of vaccination.

(Signed) HENRY HALFORD, *President*.

ALGN. FRAMPTON,<br>

THO. HUME,<br>

CHARLES BADHAM,<br>

ROBERT LLOYD, } *Censors of the Royal College of Physicians*.

EVERARD HOME, *Master of the Royal College of Surgeons*.

WILLIAM BLIZARD,<br>

HENRY CLINE, } *Governors of the Royal College of Surgeons*.

By order of the Board,

JAMES HERVEY, M.D. *Registrar*.

ART. IV.—*Case of Difficult Parturition, from the Locking of the Heads of Twins.* By JAMES ALEXANDER, jun. Surgeon, Dumferline. (Edinburgh Med. and Surg. Journal, January 1822.)

As I do not know any case similar to the following on record, I have transmitted it to you, for insertion in the Medical and Surgical Journal.

I am aware that the case which I am about to relate involves no general principle, and leads to no new or very important practical conclusion; and, consequently, that it is destitute of the highest claims to interest and attention: but if it be, as I suppose, a fact altogether new, as such it possesses, at least, a certain kind and share of interest. And, indeed, any instance, however insulated, or however unlikely to occur again, either of a new power in nature of recovery, or a new susceptibility of disease, must be deemed worthy of so much attention, at least, as to merit being preserved.

On Monday morning, the 21st of October, I was sent for to visit a woman in labour. The account I received from the midwife in attendance was, that she had been called to the person on the preceding Wednesday; but that, at this time, the dilatation of the os uteri had not commenced, and only began to take place on Sunday, though, during the three foregoing days, the pains had recurred with such regularity, and so severely, as to make the woman herself believe that she was in actual labour. That, even after this, the presentation, which was the breech, was propelled very slowly, until it advanced so far as to permit the feet to be brought down, and the body and arms extracted; but that such unexpected difficulty had occurred in delivering the head, as to render it necessary to send for some medical man to extricate it. Upon examining, I felt a head occupying the cavity of the pelvis; and, as the face was not felt in the hollow of the sacrum, and as I was anxious to complete the delivery as soon as possible, I endeavoured to turn the child, so as to bring the head into the proper position, and then to extract it. This, however, I found I could not accomplish; and, surprised at the difficulty I experienced, (which I was sure did not proceed from narrowness of the pelvis,) and puzzled by the feeling of something strange and unusual about the position of the head, I left off my endeavours at extraction, and proceeded to make a careful and accurate examination. The result of this convinced me that the head, which I felt filling the hollow of the sacrum, belonged not to the child whose body was protruded without the external parts, but to a second child, whose body still remained in utero; and that the head of the former was no farther advanced than the brim of the pelvis. Upon raising the body of the expelled child, and introducing the hand along the side of its neck, by using some force, the fingers could be pushed up betwixt it and the head of the second child, so as to ascertain that there was no connexion between them. Passing the hand along the back part of the neck, the fingers could be pushed so far as almost to touch the occipital protuberance; and, towards the left side of the pelvis, the form of the under part of the face could be distinctly recognized. In short, the face of the child, which was still in the uterus, was turned into the hollow of the other; while the face of the other (the head being at the brim,) lay towards the left side, so that the two heads were completely impacted, the lowest one obstructing the descent of the upper, while the upper, in its turn, as effectually prevented the further advancement of the body of the former. Under these circumstances, as it was perfectly impossible to push back the lowest head, it appeared to me that the only method of effecting the delivery was to puncture the nearest head immediately; and if, by this means, sufficient room should not be obtained to deliver the other head, enough would, at least, be acquired to allow the application of the scissars to it also. Before proceeding to operate, however, in a case so singular, I requested my friends, Dr. Stenhouse and Mr. Dewar, to examine the woman, and favour me with their opinion. As the opinion of these gentlemen coincided exactly with my own, I immediately punctured the lowest head. This was an easy matter, though rendered rather more difficult than it would otherwise have been, from the passage being partly occupied by the body of the other child; but, even after this had been done, it was found impossible to extract the other head, and it was therefore necessary to perforate it likewise. The only part which even yet could be felt distinctly, was the occipital protuberance, the face lying obliquely towards the left side of the pelvis; and, from the way in which the remains of the lowest head were placed with regard to the upper one, after the instrument had been conducted along the fingers to this point, (a part of the operation which required a good deal of caution,) it was necessary to depress it considerably towards the left side, as otherwise it was apt to glide over, instead of penetrating the bone. At length, after some difficulty, it was thrust into the cranium in an oblique direction, the point entering close by the foramen magnum. The brain was then broken down by the crotchet, and as much of it as possible brought away; but, even then, it was no easy matter to bring down the head, and it was only accomplished by the exertion of considerable force, both on my own part and that of the gentlemen who assisted me. After this head was extracted, the other child was easily delivered, and shortly after the placenta.

During the first and second day after delivery, no bad symptom occurred. During the second night, however, the woman was seized with a violent shivering; the pulse rose rapidly to 120; the skin became hot and dry, and the whole of the abdomen exquisitely painful and tender; and I remarked, next day, that her mouth and tongue had assumed something of the appearance which characterizes the advanced stages of typhous fever. She was immediately bled to syncope, and her bowels freely purged with calomel and neutral salts. The symptoms were, by this means, greatly relieved; but recurred on the 25th, when they were again met by a repetition of the same remedy. By persisting in the antiphlogistic mode of treatment, the pain in the belly was completely removed, and the febrile excitement very greatly diminished. The pulse, however, occasionally rose at night, though generally little above 80; and the tongue retained its dry, furred, and blackish appearance, in spite of the employment of the ordinary means to improve the state of the bowels. Still the symptoms appeared to be decidedly favourable till the 1st of November, when she was seized with another shivering; and, after it, with excruciating pain over the upper part of the sacrum and lower part of the spine, impeding motion, and aggravated by pressure on the back. About the same time, I was told that the lochia, which had disappeared after the second bleeding, had returned; and, accordingly, a very fetid bloody discharge was found flowing from the vagina. The pulse became excessively quick, and the countenance had an expression of great agony. As purgatives and diaphoretics failed in removing the pain and fever, I was obliged, though reluctantly, considering my patient's debilitated state, again to have recourse to the lancet. By one moderate bleeding, the pain was greatly alleviated; but the case now seemed perfectly hopeless. The pulse sunk in strength, but did not fail in frequency. The woman's strength declined, in spite of every attempt to support it; the countenance became collapsed; subsultus tendinum and singultus came on; and her life terminated on Wednesday the 8th of November, being the sixteenth day after delivery.

With some difficulty, permission was obtained to examine the body. On laying open the abdomen, the peritoneum, and all the abdominal viscera, (with the exception of a trifling degree of redness on some parts of the convolutions of the intestines,) appeared perfectly sound. Proceeding to the pelvis, the uterus was found reduced to nearly the size we expected to find it in ordinary cases, considering the time that had elapsed since delivery; but its substance, particularly towards the left side, felt rather softer than natural, and, upon laying it open, the whole of its inner surface, but particularly the cervix, was found thickly covered with an unhealthy secretion of a dark-brown colour and offensive smell; and, on attempting to remove it, the inner surface of the uterus, to which it adhered, was in some places torn from the subjacent parts. The appendages of the uterus, and other viscera lying in the pelvis, were healthy; but the cellular substance lining the sacrum, and lying betwixt it and the rectum, bore the marks of high inflammation of recent date, although the coats of the bowel were perfectly sound.

---

Art. V.—*On Osteo Sarcoma.* By W. Gibson, M.D. (Philadelphia Journal, No. 5.)

The older surgeons employed the terms osteo *sarcoma*, osteo *sarcosis*, and osteo *steatoma*, to designate such tumors as were formed by the irregular admixture of bony, fleshy, or fatty particles. By the moderns, *osteo sarcoma* has been retained, as more expressive than the others.

This disease may attack any of the bones, but the long bones of the extremities are commonly affected. According to Boyer,* the os innominatum is more subject to the disease than any other bone in the body. The progress of osteo sarcoma varies in different cases. Sometimes, a long-continued, deep-seated, lancinating pain, occupies some part of the bony system, long before any tumor or swelling is evident. At other times, a distinct tumor is perceptible from the first, gradually increases, and is not painful or inconvenient until it acquires considerable bulk, and takes on inflammation: the pain is then extremely severe. The form of the tumor is either smooth and circumscribed or irregular. For the most part, the general swelling is studded over with knots or protuberances, of various dimensions; the apices of which, in the advanced stages of the disease, are apt to ulcerate, and discharge a small quantity of thin fetid matter. Often, however, the whole tumor becomes enormous and extremely ponderous, without the slightest ulceration of the integuments. When examined by the touch, the tumor feels solid and incompressible; or, if any evidence exist of fluctuation, it is only at particular spots, and is even then very indistinct. Old persons are seldom subject to osteo sarcoma: I have met with two instances, however, in which the disease occurred in patients beyond the age of seventy. When young persons are attacked, a perceptible alteration is soon evinced in their general health and appearance: they become sallow, thin, and debilitated; and the bowels are alternately constipated and relaxed. Not unfrequently, a cough and tightness of respiration are constant, and very troublesome, attendants. In the advanced stages of the complaint, and especially when the tumor is large, ulcerated, and sloughy, hectic fever, and all its consequences, gradually

---

* *Traité des Maladies Chirurgicales*, tom. iii.

undermine the strength of the patient, and finally destroy him. In several instances, which have fallen under my notice, the patient has died from confirmed phthisis pulmonalis.

When the structure of osteo sarcoma is examined by dissection, several interesting circumstances are developed. The integuments being raised, the muscles and tendons are found removed from their natural situation, and spread out and thinned to such an extent, as to cover a much larger surface than they usually occupy. The vessels and nerves of the part are also raised, and made to approach the surface of the tumor: to this surface the periosteum will be found closely to adhere, and to give a firm, dense, and pearl-coloured covering, which is with great difficulty separated from the diseased bone. The bone itself, in immediate contact with its investing membrane, will be found smooth on the surface, and either uniform and regular, or else disposed in lobulated masses of different forms and sizes. Sometimes these are extremely regular, of a rounded form, and resemble very much a cluster of grapes. At other times, several large masses are joined together, and present the appearance of an artichoke or protuberant potato. When the bony texture of these tumors is cut, forcibly separated, or crushed, a number of irregular cells are brought into view, containing either a thick, cheesy, lardaceous, medullary matter, or else a gelatinous semi-transparent fluid, which oozes out of its own accord, or can be removed by mechanical means, or by maceration, leaving the sides of the cavities lined by a very fine and delicate membrane. The morbid tissue of bone will then be found to consist of innumerable spicula, disposed in endless variety of ramifications, and shooting out into fantastic forms, resembling some species of coral, or assuming the shape of certain vegetable productions. I have in my possession a very fine specimen of osteo sarcoma, of several pounds weight, taken from the upper-jaw of an ox, and presented to me by a very intelligent young physician, Dr. Townsend, of Maryland; to whom my cabinet is indebted for other valuable contributions. In this specimen, the cells which I have described, and the arrangement of the bony spicula, are uncommonly well displayed, owing to the magnitude of the tumor, and the original texture of the bone upon which it is reared.

The origin of osteo sarcoma is involved in great obscurity. It appears, sometimes, as an hereditary disease; and, as such, has been transmitted, in succession, to numerous individuals of the same family. A very remarkable case of this kind is recorded by Boyer, in which the father, brothers, sisters, nephews, and children, of a woman, thirty years of age, and who otherwise enjoyed good health, had, from their earliest infancy, bony tumors on the tibia. The patient herself had similar tumors on both tibiæ, on the left humerus, and on the middle of the left thigh. All remained stationary, and were of small size, except the one on the thigh, which gradually increased, became particularly large and painful after her marriage, and, finally, increased to such an extent as to weigh twenty-one pounds, and render amputation necessary. Upon dissection, all the characters of osteo sarcoma were distinctly marked. In many instances, this disease has been decidedly traced to a blow, to a jump from a height, to fracture, and other external injury; but there is great reason to believe, in most instances, that it is connected with some constitutional affection, since we meet with many cases in which, after removal of the tumor or amputation of a limb, other parts of the body have been attacked in a similar manner, or else the patient has been carried off in a short time by some disturbance of the vital organs. In several cases presented to my notice, patients have undergone operations for osteo sarcoma, and have so far recovered, in a short time, as apparently to enjoy excellent health; when, suddenly, their strength has declined, hectic fever has supervened, a pain in the breast, with cough and purulent expectoration, has taken place, and death has soon followed from phthisis pulmonalis. A case is recorded by Dr. Baillie, in his Morbid Anatomy, of a person who had a very large bony tumor formed around one of his knees: this was removed, at St. George Hospital, by Mr. Walker, by amputation of the limb. Very soon after, a difficulty of breathing began, occasioned by part of the lungs being converted into bone, and by a very considerable deposition of bony matter on the inside of several of the ribs, which caused the patient's death.* These circumstances would seem to favour the idea suggested by Boyer,† Richerand,‡ Callisen,§ and some other writers, that osteo sarcoma is, in reality, a cancerous affection of the bony tissue; and, as such, may give rise to all the consequences which are liable to result from a similar disease of the soft parts. It does not follow, however, admitting this view of the subject to be correct, that the disease is always so deeply engrafted upon the constitution as necessarily to give rise, after extirpation or amputation, to a similar affection of the pulmonary system, or any other of the vital organs. On the contrary, from the favourable result of several operations for the removal of osteo sarcoma, there is reason to believe that, when resorted to in time, the disease may be as successfully extirpated as cancer of the soft parts; but, like that disease, if permitted to remain until the system is extensively

* Wilson's *Lectures on the Bones and Joints,* p. 274.

† *Maladies Chirurgicales,* tom. iii. p. 587.

‡ *Nosographie Chirurgicale,* tom. iii. p. 121.

§ *Systema Chirurgiæ Hodiernæ,* vol. ii. p. 204.

contaminated, may prove equally fatal. How far scrofula can be considered as giving rise to, or as connected with, osteo sarcoma, as it is known to be with some other affections of the bones, remains yet to be determined.

*Treatment.*—Before an osteo-sarcomatous tumor has attained a large size, it may sometimes be removed by local and constitutional remedies, without the aid of an operation. Leeches, applied to the part itself, or its vicinity, will be found very useful. Blisters, also, often repeated, and kept up by savin cerate, will prove still more beneficial. As a constitutional remedy, Mr. Cooper* has lately extolled the exhibition of oxymuriate of mercury, combined with the compound decoction of sarsaparilla; a medicine which has long been used in France and in this country, with the happiest effects, in the treatment of various diseases, especially chronic ulcerations and tumors of different kinds. Low diet, conjoined with purgatives, must likewise be had recourse to: and, perhaps, moderate pressure, steadily applied to the tumor, may prove useful. When these remedies fail, an operation will become necessary. In many instances, nothing less than amputation will answer, inasmuch as the whole circumference of a bone is involved in the disease. Great care should be taken, therefore, to ascertain the extent and connexions of the tumor, as, without just views in this respect, much mischief, and unnecessary pain and hazard to the patient, may result. Mr. John Bell has related the case of a labourer, forty years of age, who had a tumor of enormous size, and of anomalous character, partly cartilaginous and partly solid, occupying two-thirds of the fore-arm, from the wrist upwards. The hand was sound; the fingers and wrist could be easily bent; and the tumor seemed to move so freely, that a surgeon of skill and learning was induced to undertake its extirpation, in hopes of saving the hand and joint. "The poor man having willingly assented to any operation, however lingering or painful, which might save his hand, the dissection was carried all around the tumor, and into its central parts, before the surgeons present were undeceived. As the radius turns vertically like a spoke or spindle, it turns without any apparent motion, except in the parts connected with its lower end; the hand turns freely along with the radius, so that we never suspect, till we become acquainted with anatomy, that it is by the spoke-like motions of the radius that the hand moves: it seems movable in itself, by its own immediate joints. This tumor, in like manner, moved easily, could be turned upwards and downwards; so that the surgeon never once suspected that the motion was in the radius, or that the tumor was fixed, and made a part

---

* *Surgical Essays*, p. 183.

of the bone. It seemed moveable; and, doubting, he began to extirpate it, by drawing a long incision round its root, on the side of the ulna; but, finding it difficult, with this limited incision, to dissect the tumor, he prolonged the incision, continuing it over the back of the hand to the knuckles, in the direction of the extensor tendons. He then dissected more freely, and continued separating the skin from the tumor, till he came to a thick and solid sac, which seemed to consist of the muscular fibres and aponeurosis of the pronator quadratus muscle. He continued this dissection, separating this thick and solid sac from the interosseous ligament, till he could go no further. Finding that it terminated in a solid and osseous basis, he now plunged intrepidly into the heart of the tumor. In cutting into the heart of the tumor, he found that he had opened a very large sac, not firm only, but osseous: but still, as he was penetrating into the tumor at one side, he continued still unsuspicious, and persevered in dissecting away what he imagined to be a common tendinous sac, ossified only at certain points. He made, thus, a large opening into the tumor,—felt its cavity full of loose and fatty bodies,—pushed his finger under the extensor tendons into the deepest part of the sac,—began to hook out the fatty tubercles with his fingers,—and, at last, baling it out with his hand, hooking with his finger, and catching the fatty masses in his palm, he so far emptied the cavity as to be able to search with his fingers in every direction; and then he found, to his utter confusion, the ball of the carpus formed by the scaphoid and lunated bones, at the bottom of the cavity, bare. He was now, for the first time, undeceived, and knew what sort of disease he had to contend with: he was now conscious that the radius was diseased,—the joint destroyed,—the original bone ulcerated. He felt distinctly that the ball of the carpal bone, originally opposed to the lower end of the radius, was now, by the destruction of the radius, left naked; and, in fine, that the wrist was irrecoverably ruined. There was no going on with the operation, and no stopping here: he therefore explained to the patient, who had borne this severe and long-protracted dissection with great composure, the necessity of amputating his hand, which he submitted to with equal resignation."*

The above case is calculated to show, in a striking manner, with how little prospect of success we can undertake to remove an osteo sarcoma of any magnitude, involving the whole circumference of a bone: but it still remains a question, whether such a tumor, while in its incipient state, of small size, and seated on one of the bones of the fore-arm or leg, might not be

* *Principles of Surgery*, vol. iii. p. 64.

successfully extirpated by sawing through the sound bone above and below its margins. The extremities of the diseased bone would eventually, perhaps, be filled up, or connected by adventitious ligaments, and so much support afterwards given by the sound bone, which would act as a splint, as to render the limb sufficiently useful for most purposes. Much, however, must necessarily depend, in such a case, upon the precise situation of the tumor; if closely connected with a joint, the operation would be attended with risk, and, in the end, would, perhaps, prove unsuccessful, or give rise to subsequent amputation. It will appear, from what has been said, that the treatment of osteo sarcoma must be different, in many respects, from that of exostosis; that we cannot extirpate the former, with the same success as the latter; and that, in many instances, owing to constitutional disturbance, all our operations will prove unavailing. This remark will even apply to amputation; for it has been decidedly ascertained, that the lungs, and other important internal organs, have been attacked, in a very short time, after the removal of a limb affected with the disease. To guard against such unpleasant consequences, our only resource is to continue the constitutional treatment recommended, after the operation, and to substitute an issue in the vicinity of the part from which the tumor has been removed.

Experience has, long ago, taught me that, without precautions of this kind, the patient's chance of recovery will be very much lessened. It appears, too, that the very ample and extended observations of Mr. Cooper have taught him the same lesson, if we may judge from the hint contained in his essay on "Fungous Exostosis;" a term apparently employed by him to designate osteo sarcoma. "The operation of amputation," says he, "after constitutional means have been employed, and the continuance of these constitutional means after the operation, hold out the chief hope of safety; for amputation without these will do no more than to avert the blow for a season."* As a local remedy for osteo sarcoma, Mr. Cooper was induced to try the effect of cutting off the supply of blood from the tumor, by tying the arteries which supplied it. The operation was accordingly performed in two cases, but without success; the current of blood being temporarily diminished, but returning in a short time with its accustomed force.

---

* *Surgical Essays*, p. 186.

